# 4-(1,3-Thia­zolidin-2-yl)phenol

**DOI:** 10.1107/S1600536809042135

**Published:** 2009-10-23

**Authors:** Xue-Mei Yang

**Affiliations:** a Department of Chemistry, Guangdong Medical College, Dongguan 523808, People’s Republic of China

## Abstract

In the title compound, C_9_H_11_NOS, the thia­zolidinyl ring is almost perpendicular to the phenyl ring with N—C—C—C torsion angles of 71.7 (2) and 107.1 (2)°. In the crystal, mol­ecules are connected *via* N—H⋯O and O—H⋯N hydrogen bonds, forming layers.

## Related literature

For the cyclization of 2-amino-ethanthiol Schiff bases, see: Al-Sayyab *et al.* (1968 ▶); Stacy & Strong (1967 ▶); Thompson & Busch (1964 ▶).
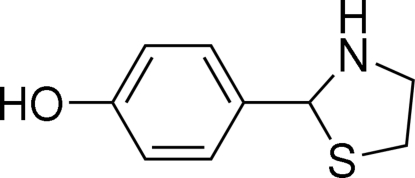


## Experimental

### 

#### Crystal data

C_9_H_11_NOS
*M* *_r_* = 181.25
Orthorhombic, 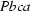

*a* = 12.3638 (6) Å
*b* = 8.9683 (5) Å
*c* = 15.8249 (8) Å
*V* = 1754.7 (2) Å^3^
*Z* = 8
Mo *K*α radiation
μ = 0.32 mm^−1^
*T* = 173 K
0.47 × 0.45 × 0.16 mm

#### Data collection

Bruker SMART 1000 CCD diffractometer
Absorption correction: multi-scan (*SADABS*; Sheldrick, 2004 ▶) *T*_min_ = 0.865, *T*_max_ = 0.951
9635 measured reflections
1919 independent reflections
1615 reflections with *I* > 2σ(*I*)
*R*_int_ = 0.022

#### Refinement

*R*[*F* ^2^ > 2σ(*F* ^2^)] = 0.037
*wR*(*F* ^2^) = 0.105
*S* = 1.07
1919 reflections
115 parameters
H atoms treated by a mixture of independent and constrained refinement
Δρ_max_ = 0.37 e Å^−3^
Δρ_min_ = −0.17 e Å^−3^

### 

Data collection: *SMART* (Bruker, 2001 ▶); cell refinement: *SAINT-Plus* (Bruker, 2003 ▶); data reduction: *SAINT-Plus*; program(s) used to solve structure: *SHELXTL* (Sheldrick, 2008 ▶); program(s) used to refine structure: *SHELXTL*; molecular graphics: *SHELXTL*; software used to prepare material for publication: *SHELXTL*.

## Supplementary Material

Crystal structure: contains datablocks global, I. DOI: 10.1107/S1600536809042135/im2144sup1.cif

Structure factors: contains datablocks I. DOI: 10.1107/S1600536809042135/im2144Isup2.hkl

Additional supplementary materials:  crystallographic information; 3D view; checkCIF report

## Figures and Tables

**Table 1:** Hydrogen-bond geometry (Å, °)

*D*—H⋯*A*	*D*—H	H⋯*A*	*D*⋯*A*	*D*—H⋯*A*
N1—H1⋯O1^i^	0.85 (2)	2.28 (2)	3.073 (2)	156 (2)
O1—H1*A*⋯N1^ii^	0.82 (2)	1.91 (2)	2.713 (2)	164 (2)

Symmetry codes: (i) 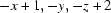; (ii) 
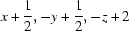
.

**Fig. 1.**
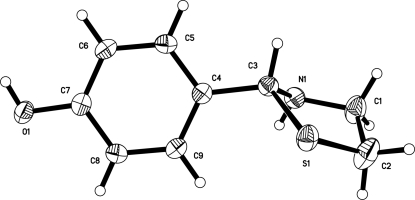
The molecular structure with thermal ellipsoids drawn at the 30% probability level.

**Fig. 2.**
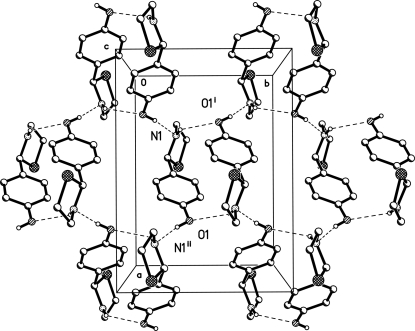
Crystal lattice along c axis. H atoms not involved in hydrogen bonds have been omitted for clarity.

## supplementary crystallographic information

### Comment

In our search for a new synthetic route to imipenem, a carbapenem antibiotic, we got a thiazolidine compound from a reaction of *p*-hydroxybenzaldehyde with 2-amino-ethanthiol, despite of our initial plan to prepare a Schiff base compound. This is consistent with reports that the 2-amino-ethanthiol Schiff base compounds can undergo intromolecular cyclization to form thiazolidines (Al-Sayyab *et al.*, 1968; Thompson & Busch, 1964; Stacy & Strong, 1967).

In the molecular sturcture (Fig. 1), as it is expected the thiazolidinyl ring is not planar, showing a N(1)—C(1)—C(2)—S(1) torsion angle of -33.7 (2)°. Furthermore, the thiazolidinyl ring is almost perpendicular to the phenyl ring, with torsion angles N(1)—C(3)—C(4)—C(9) of 71.7 (2)° and N(1)—C(3)—C(4)—C(5) of 107.1 (2)°. In Fig. 1 the chiral center C(3) adopts *R* configuation. Nevertheless, due to space group symmetry a reacemate has been formed and both enantiomers are present in the crystal structure.

In the crystal structure two adjacent molecules are connected *via* N—H···O and O—H···N hydrogen bonds to form centrosymmetric molecule pairs. These pairs are further linked by additional N—H···O and O—H···N intermolecular hydrogen bonds leading to the observed layered supramolecular (Fig. 2).

### Experimental

2-Amino-ethanthiol 0.77 g (0.001 mol) was mixed with *p*-hydroxybenzaldehyde 1.22 g (0.001 mol) in ethanol (10 ml) and the mixture refluxed for 2 h. The solvent was evaporated to dryness under reduced pressure and the remaining residue recrystallized from ethanol to afford 1.5 g of yellow block crystals. (Yield 85%). Crystals suitable for X-ray diffraction were obtained by slow evaporation of an ethanolic solution. Spectroscopic analysis: ^1^H NMR (DMSO-d_6_, δ, p.p.m.): 2.75–2.90 (m, 2H), 2.85–3.05 (m, 2H), 3.50 (m, 1H), 5.35 (s, 1H), 6.70 (d, 2H), 7.25 (d, 2H), 9.35 (s, 1H); elemental analysis, calculated for C_9_H_11_NOS: C, 59.67; H, 6.08; N, 7.73; found: C, 59.33; H, 5.93; N 7.41%.

### Refinement

All H-atoms were positioned geometrically and refined using a riding model with d(C—H) = 0.95 Å, *U*_iso_=1.2*U*_eq_ (C) for aromatic 1.00 Å, *U*_iso_ = 1.2*U*_eq_ (C) for CH, 0.99 Å, *U*_iso_ = 1.2*U*_eq_ (C) for CH_2_ and 0.88 Å, *U*_iso_ = 1.5*U*_eq_ (N) for the NH atoms.

### Crystal data

**Table:**

C_9_H_11_NOS	*F*(000) = 768
*M**_r_* = 181.25	*D*_x_ = 1.372 Mg m^−^^3^
Orthorhombic, *P**b**c**a*	Mo *K*α radiation, λ = 0.71073 Å
Hall symbol: -P 2ac 2ab	Cell parameters from 4931 reflections
*a* = 12.3638 (6) Å	θ = 2.6–27.0°
*b* = 8.9683 (5) Å	µ = 0.32 mm^−^^1^
*c* = 15.8249 (8) Å	*T* = 173 K
*V* = 1754.7 (2) Å^3^	Block, colorless
*Z* = 8	0.47 × 0.45 × 0.16 mm

### Data collection

**Table:**

Bruker SMART 1000 CCD diffractometer	1919 independent reflections
Radiation source: fine-focus sealed tube	1615 reflections with *I* > 2σ(*I*)
graphite	*R*_int_ = 0.022
ω scans	θ_max_ = 27.0°, θ_min_ = 2.6°
Absorption correction: multi-scan (*SADABS*; Sheldrick, 2004)	*h* = −15→15
*T*_min_ = 0.865, *T*_max_ = 0.951	*k* = −11→8
9635 measured reflections	*l* = −20→17

### Refinement

**Table:**

Refinement on *F*^2^	Primary atom site location: structure-invariant direct methods
Least-squares matrix: full	Secondary atom site location: difference Fourier map
*R*[*F*^2^ > 2σ(*F*^2^)] = 0.037	Hydrogen site location: inferred from neighbouring sites
*wR*(*F*^2^) = 0.105	H atoms treated by a mixture of independent and constrained refinement
*S* = 1.07	*w* = 1/[σ^2^(*F*_o_^2^) + (0.0611*P*)^2^ + 0.7197*P*] where *P* = (*F*_o_^2^ + 2*F*_c_^2^)/3
1919 reflections	(Δ/σ)_max_ < 0.001
115 parameters	Δρ_max_ = 0.37 e Å^−^^3^
0 restraints	Δρ_min_ = −0.17 e Å^−^^3^

### Special details

**Table:**

Geometry. All e.s.d.'s (except the e.s.d. in the dihedral angle between two l.s. planes) are estimated using the full covariance matrix. The cell e.s.d.'s are taken into account individually in the estimation of e.s.d.'s in distances, angles and torsion angles; correlations between e.s.d.'s in cell parameters are only used when they are defined by crystal symmetry. An approximate (isotropic) treatment of cell e.s.d.'s is used for estimating e.s.d.'s involving l.s. planes.
Refinement. Refinement of *F*^2^ against ALL reflections. The weighted *R*-factor *wR* and goodness of fit *S* are based on *F*^2^, conventional *R*-factors *R* are based on *F*, with *F* set to zero for negative *F*^2^. The threshold expression of *F*^2^ > σ(*F*^2^) is used only for calculating *R*-factors(gt) *etc*. and is not relevant to the choice of reflections for refinement. *R*-factors based on *F*^2^ are statistically about twice as large as those based on *F*, and *R*- factors based on ALL data will be even larger.

### Fractional atomic coordinates and isotropic or equivalent isotropic displacement parameters (Å^2^)

**Table:**

	*x*	*y*	*z*	*U*_iso_*/*U*_eq_	
S1	0.47979 (3)	0.18010 (5)	0.70817 (2)	0.02679 (16)	
C1	0.27121 (14)	0.1895 (2)	0.74816 (11)	0.0355 (4)	
H1B	0.2501	0.2924	0.7327	0.043*	
H1C	0.2046	0.1288	0.7530	0.043*	
C2	0.34426 (15)	0.1245 (3)	0.67981 (12)	0.0417 (5)	
H2A	0.3245	0.1646	0.6236	0.050*	
H2B	0.3381	0.0145	0.6783	0.050*	
C3	0.43704 (12)	0.24880 (18)	0.81446 (9)	0.0217 (3)	
H3	0.4323	0.3600	0.8119	0.026*	
C4	0.51646 (12)	0.20804 (17)	0.88292 (9)	0.0209 (3)	
C5	0.55139 (13)	0.31669 (17)	0.93977 (10)	0.0235 (3)	
H5	0.5255	0.4159	0.9342	0.028*	
C6	0.62307 (13)	0.28297 (18)	1.00422 (10)	0.0248 (4)	
H6	0.6461	0.3587	1.0421	0.030*	
C7	0.66115 (13)	0.13804 (18)	1.01328 (9)	0.0227 (3)	
C8	0.62657 (13)	0.02820 (18)	0.95705 (10)	0.0239 (3)	
H8	0.6521	−0.0712	0.9630	0.029*	
C9	0.55519 (12)	0.06344 (18)	0.89265 (10)	0.0229 (3)	
H9	0.5323	−0.0122	0.8546	0.027*	
N1	0.32818 (11)	0.19058 (16)	0.82922 (9)	0.0249 (3)	
H1	0.3303 (16)	0.103 (3)	0.8493 (12)	0.030*	
O1	0.73103 (10)	0.09658 (14)	1.07553 (7)	0.0302 (3)	
H1A	0.7567 (19)	0.172 (3)	1.0971 (13)	0.036*	

### Atomic displacement parameters (Å^2^)

**Table:**

	*U*^11^	*U*^22^	*U*^33^	*U*^12^	*U*^13^	*U*^23^
S1	0.0253 (2)	0.0357 (3)	0.0194 (2)	0.00118 (16)	0.00148 (14)	−0.00048 (16)
C1	0.0239 (9)	0.0512 (12)	0.0313 (9)	0.0019 (8)	−0.0045 (7)	−0.0055 (8)
C2	0.0308 (9)	0.0616 (14)	0.0326 (9)	−0.0052 (9)	−0.0028 (8)	−0.0139 (9)
C3	0.0218 (7)	0.0221 (8)	0.0213 (7)	0.0008 (6)	0.0012 (6)	−0.0004 (6)
C4	0.0213 (7)	0.0232 (8)	0.0182 (7)	−0.0019 (6)	0.0023 (6)	0.0005 (6)
C5	0.0265 (8)	0.0188 (7)	0.0252 (8)	−0.0003 (6)	0.0013 (6)	−0.0004 (6)
C6	0.0283 (8)	0.0229 (8)	0.0233 (7)	−0.0042 (6)	−0.0009 (6)	−0.0046 (6)
C7	0.0214 (7)	0.0271 (8)	0.0197 (7)	−0.0034 (6)	0.0012 (6)	0.0013 (6)
C8	0.0259 (8)	0.0204 (7)	0.0254 (8)	0.0011 (6)	0.0009 (6)	−0.0003 (6)
C9	0.0236 (7)	0.0233 (8)	0.0218 (7)	−0.0033 (6)	0.0008 (6)	−0.0027 (6)
N1	0.0219 (7)	0.0266 (7)	0.0262 (7)	0.0003 (5)	0.0013 (5)	0.0001 (6)
O1	0.0337 (7)	0.0275 (6)	0.0293 (6)	−0.0026 (5)	−0.0117 (5)	−0.0012 (5)

### Geometric parameters (Å, °)

**Table:**

S1—C2	1.8049 (19)	C4—C5	1.395 (2)
S1—C3	1.8676 (15)	C5—C6	1.385 (2)
C1—N1	1.463 (2)	C5—H5	0.9500
C1—C2	1.525 (3)	C6—C7	1.390 (2)
C1—H1B	0.9900	C6—H6	0.9500
C1—H1C	0.9900	C7—O1	1.3620 (19)
C2—H2A	0.9900	C7—C8	1.395 (2)
C2—H2B	0.9900	C8—C9	1.385 (2)
C3—N1	1.462 (2)	C8—H8	0.9500
C3—C4	1.507 (2)	C9—H9	0.9500
C3—H3	1.0000	N1—H1	0.85 (2)
C4—C9	1.391 (2)	O1—H1A	0.82 (2)
			
C2—S1—C3	93.00 (8)	C5—C4—C3	119.73 (14)
N1—C1—C2	109.83 (14)	C6—C5—C4	121.38 (15)
N1—C1—H1B	109.7	C6—C5—H5	119.3
C2—C1—H1B	109.7	C4—C5—H5	119.3
N1—C1—H1C	109.7	C5—C6—C7	119.81 (14)
C2—C1—H1C	109.7	C5—C6—H6	120.1
H1B—C1—H1C	108.2	C7—C6—H6	120.1
C1—C2—S1	105.55 (12)	O1—C7—C6	123.01 (14)
C1—C2—H2A	110.6	O1—C7—C8	117.59 (14)
S1—C2—H2A	110.6	C6—C7—C8	119.40 (14)
C1—C2—H2B	110.6	C9—C8—C7	120.25 (15)
S1—C2—H2B	110.6	C9—C8—H8	119.9
H2A—C2—H2B	108.8	C7—C8—H8	119.9
N1—C3—C4	113.46 (13)	C8—C9—C4	120.91 (14)
N1—C3—S1	106.65 (10)	C8—C9—H9	119.5
C4—C3—S1	112.52 (11)	C4—C9—H9	119.5
N1—C3—H3	108.0	C3—N1—C1	107.78 (13)
C4—C3—H3	108.0	C3—N1—H1	111.2 (14)
S1—C3—H3	108.0	C1—N1—H1	109.8 (13)
C9—C4—C5	118.25 (14)	C7—O1—H1A	108.7 (15)
C9—C4—C3	122.01 (14)		
			
N1—C1—C2—S1	−33.3 (2)	C5—C6—C7—O1	−179.46 (15)
C3—S1—C2—C1	10.32 (15)	C5—C6—C7—C8	0.1 (2)
C2—S1—C3—N1	14.01 (13)	O1—C7—C8—C9	179.74 (14)
C2—S1—C3—C4	139.03 (13)	C6—C7—C8—C9	0.2 (2)
N1—C3—C4—C9	71.65 (19)	C7—C8—C9—C4	−0.2 (2)
S1—C3—C4—C9	−49.55 (18)	C5—C4—C9—C8	0.0 (2)
N1—C3—C4—C5	−107.07 (17)	C3—C4—C9—C8	−178.77 (14)
S1—C3—C4—C5	131.73 (13)	C4—C3—N1—C1	−159.92 (14)
C9—C4—C5—C6	0.3 (2)	S1—C3—N1—C1	−35.47 (15)
C3—C4—C5—C6	179.05 (14)	C2—C1—N1—C3	45.7 (2)
C4—C5—C6—C7	−0.3 (2)		

### Hydrogen-bond geometry (Å, °)

**Table:**

*D*—H···*A*	*D*—H	H···*A*	*D*···*A*	*D*—H···*A*
N1—H1···O1^i^	0.85 (2)	2.28 (2)	3.073 (2)	156 (2)
O1—H1A···N1^ii^	0.82 (2)	1.91 (2)	2.713 (2)	164 (2)

Symmetry codes: (i) −*x*+1, −*y*, −*z*+2; (ii) *x*+1/2, −*y*+1/2, −*z*+2.
